# From unified to differentiated materials: generative AI–supported adaptation of EAP reading materials

**DOI:** 10.3389/fpsyg.2026.1887565

**Published:** 2026-08-13

**Authors:** Xuewei Gao

**Affiliations:** School of General Education (Public Art Education Center), Xihang University, Xi'an, Shaanxi, China

**Keywords:** academic fidelity, English for Academic Purposes, GenAI-supported adaptation, structural complexity, text-embedded support

## Abstract

**Introduction:**

Generative artificial intelligence (GenAI) can adapt English for Academic Purposes (EAP) reading materials by rewriting passages, adding support, or combining both. This study examined whether proficiency-sensitive GenAI adaptation chiefly changed passage-level structural complexity or text-embedded functional support while preserving academic fidelity.

**Methods:**

A role-prompted workflow combined barrier analysis, adaptation, fidelity checking, and validation. In a 3 × 3 between-subjects design (*N* = 135; *n* = 15 per cell), proficiency level (low, intermediate, and high) was crossed with material condition (original, unified-AI, and differentiated-AI). Three EAP instructors evaluated 15 anonymized material versions. Automated structural-complexity indicators, leave-one-out discriminant analysis, within-proficiency planned contrasts, and omnibus outcome models were used to assess material and learner outcomes.

**Results:**

The instructors rated the materials favorably for academic fidelity (*M* = 4.24), proficiency appropriateness (*M* = 4.36), and teachability (*M* = 4.31), with acceptable inter-rater reliability, *ICC*(2, *k*) = 0.84. Automated structural-complexity indicators showed limited separation ($\eta_{p}^{2}=0.043$), and leave-one-out discriminant analysis classified the intended proficiency labels at 11.1%, below the 33.3% balanced-task benchmark. Material differences were clearest in functional support, including glosses, sentence unpacking, rhetorical cues, claim-evidence notes, and critical prompts. Differentiated-AI exceeded unified-AI most strongly among high-proficiency learners (*d* = 1.40), followed by a moderate advantage among low-proficiency learners (*d* = 0.60) and a small difference among intermediate learners (*d* = 0.16). The omnibus reading-comprehension interaction identified this heterogeneity, F_(4, 126)_ = 7.43, *p* < 0.001, and $\eta_{p}^{2}=0.191$. Supplementary process estimates were descriptive because the process indicators and outcomes were collected in the same session. Immediate unsupported application did not differ by material condition or interaction.

**Discussion:**

The findings locate GenAI-supported differentiation primarily in proficiency-specific support-layer design rather than broad changes in passage-level structural complexity. The strongest learner evidence came from comparisons conducted within the same proficiency level.

## 1 Introduction

EAP reading is difficult because academic language and academic argument operate at the same time. Learners encounter technical vocabulary, nominalization, long clause chains, stance markers, hedging, citation relations, and claim–evidence organization within the same passage. Reading, therefore, requires more than sentence-level comprehension: learners must also see how writers qualify claims, connect evidence to warrants, signal disciplinary caution, and position knowledge within a genre (Swales, 2004; Hyland, 2006, 2019). A material that helps learners only by reducing lexical and syntactic difficulty may leave the academic work of the passage untouched.

Mixed-proficiency EAP classrooms sharpen this design problem. Low-proficiency learners often struggle to enter the passage because vocabulary, syntax, and working-memory load block local meaning. Intermediate learners may understand many sentences yet lose paragraph function, transition, and evidence relations. High-proficiency learners often need less help with access and more help with stance evaluation, evidence judgment, counterargument, and disciplinary response. Unified reading materials are convenient for teaching and assessment. They also compress these different barriers into one easier–harder scale (Grabe, 2009; Nation, 2013). Proficiency-sensitive adaptation in this article means matching the type and density of support to these different barriers and evaluating that match through material ratings, learner outcomes, and process-related indicators.

Text simplification can help learners enter a source passage, and recent controllable-simplification work has shown how target level, fluency, correctness, and meaning preservation can guide rewritten texts (Agrawal and Carpuat, 2023; Bahrainian et al., 2024; Maddela et al., 2023). EAP adaptation also has to protect hedging, citation relations, disciplinary terminology, and argumentative structure, since removing these features can make a passage easier to read and less useful for academic reading instruction. Academic fidelity names the part of adaptation that protects the source text's disciplinary meaning, terminology, stance markers, evidence relations, and genre function. In the materials examined here, academic fidelity appears in retained technical terms, preserved qualification, intact claim–evidence links, and prompts that keep learners working with the author's argument. It is assessed through expert ratings of the generated versions.

Two material layers organize the analysis: structural complexity and functional support. Structural complexity refers to passage-level linguistic features that can be measured automatically, including lexical density, sentence length, clauses per sentence, academic vocabulary coverage, cohesion markers, and hedging markers. Functional support refers to assistance that changes how learners work with the passage: glosses, sentence unpacking, rhetorical labels, paragraph-function notes, claim–evidence cues, and critical prompts. Text-embedded support is the visible material form of that functional support, printed into or around the reading passage. A differentiated version may leave the main passage structurally similar to the source text while changing the reading task by guiding lexical access, discourse mapping, or evaluative response.

For this problem, GenAI is useful when it helps teachers generate support layers targeted to different barriers. Recent language-education work has treated GenAI as a tool for feedback, writing support, dialogue, and materials development, with parallel concerns about overreliance, hallucinated content, and teacher oversight (Hockly, 2023; Kohnke et al., 2023; Kasneci et al., 2023; Fang and Han, 2025). Fluent paraphrases alone leave the central design problem unresolved. Generated materials may add unsupported content, flatten stance, over-simplify disciplinary reasoning, or hide consequential prompt decisions. The role-prompted GenAI workflow examined here addresses that risk by separating generation into four auditable modules. Barrier analysis takes a source text and target proficiency profile and outputs likely reading obstacles; adaptation outputs a material version with glosses, cues, or prompts; academic-fidelity checking examines terminology, stance, hedging, and argument structure; validation checks level fit, coherence, and teachability.

Recent reviews of GenAI and conversational AI in language education show growing empirical activity, yet reading, material validation, prompt transparency, and short-term task evidence remain less visible than writing support and self-report perceptions (Lai and Lee, 2024; Lee et al., 2025; Li et al., 2025; Wang et al., 2025). The empirical design addresses four connected questions: whether the role-prompted GenAI workflow can produce differentiated EAP materials that expert reviewers judge academically faithful, level-appropriate, and teachable; whether the low-, intermediate-, and high-proficiency versions differ in passage-level structural complexity or chiefly in the functional support layer; whether proficiency-specific support adds value beyond a single AI-adapted version; and whether immediate unsupported application, a new unmodified passage completed without glosses, labels, or prompts after the main reading task, shows lower short-term unassisted performance after supported reading. The learner-outcome analysis gives priority to planned contrasts within each proficiency stratum, comparing differentiated-AI with unified-AI while holding proficiency constant. The omnibus Proficiency × Material model describes heterogeneity across strata, and the planned contrasts provide the cleaner evidence for whether differentiated support adds value over a unified AI-adapted material.

## 2 Related work

### 2.1 EAP reading, genre, and text-embedded support

EAP reading adaptation has to protect the academic work carried by the source text. Genre theory treats academic texts as purposeful discourse, and research on metadiscourse and stance shows that writers use hedging, citation, self-positioning, and rhetorical organization to manage disciplinary claims (Swales, 2004; Hyland, 2006, 2019). A learner who misses these features may understand a sentence and still miss how the passage builds knowledge. For this reason, academic fidelity in EAP materials is tied to claim–evidence relations, paragraph function, stance, hedging, terminology, and disciplinary meaning.

Text-embedded support gives learners access to these discourse features at the point of reading. Academic literacy research frames support as entry into disciplinary expectations, and EAP scholarship explains why conventions such as evidence use, qualification, and genre function often need direct attention (Wingate, 2015; Hyland, 2018). Scaffolding theory adds the criterion of learner readiness and task fit (Wood et al., 1976; Vygotsky, 1978; Reynolds, 2017). The present study uses scaffolding in a restricted sense: fixed, text-embedded, task-bound support printed into the material, including glosses, rhetorical labels, paragraph cues, claim–evidence notes, and critical prompts. The expert-rating rubric evaluates whether these supports preserve academic fidelity, fit the intended proficiency level, and remain teachable.

Reading theory explains why the same academic passage may require different supports. Construction–integration accounts link local processing to the building of a coherent text representation, and cognitive-load research explains how vocabulary, syntax, and task demands can consume resources before discourse interpretation begins (Kintsch, 1998; Paas et al., 2003; Sweller et al., 2011). These traditions justify a barrier-based mapping: lexical and syntactic access for low-proficiency readers, discourse organization for intermediate readers, and stance or evidence evaluation for high-proficiency readers.

### 2.2 Text adaptation and complexity evaluation

Text adaptation research offers tools for changing the main passage itself. Controllable simplification specifies operations such as lexical substitution, sentence splitting, syntactic rewriting, and paraphrasing. Grade-specific and adaptive simplification work shows how target level, fluency, correctness, and meaning preservation can guide rewriting (Agrawal and Carpuat, 2023; Bahrainian et al., 2024). Evaluation work in simplification also stresses the need for metrics that correspond to human judgments when generated text must remain meaningful and useful (Maddela et al., 2023).

Automated complexity metrics are well-suited to passage-level structure. Coh-Metrix examines cohesion and language features beyond sentence length (Graesser et al., 2004). Lexical-diversity measures, syntactic-complexity measures, and readability research add indicators for lexical density, clausal demand, and simplification evaluation (McCarthy and Jarvis, 2010; Lu, 2010; Vajjala and Meurers, 2014). In the present study, lexical density, average sentence length, clauses per sentence, academic vocabulary coverage, cohesion markers, and hedging markers operationalize structural complexity. These metrics answer whether the differentiated versions changed the main passage in measurable linguistic form.

The same metrics do not evaluate the instructional function of glosses, rhetorical labels, paragraph-function notes, or critical prompts. Those features sit in the support layer and alter the reader's task without changing the sentence structure of the passage. The separation between structural complexity and functional support, therefore, determines how the material evidence is interpreted: automated metrics locate passage-level change, while expert ratings judge whether the support layer preserves academic fidelity and can be used pedagogically.

### 2.3 Generative AI in language education

GenAI has been used in language education for feedback, writing support, content generation, dialogue practice, and teacher preparation (Godwin-Jones, 2023; Barrot, 2023; Warschauer et al., 2023). For EAP reading material development, its value lies in generating alternative support designs from the same source passage under explicit audience and instructional-role prompts. A useful output preserves terminology, stance, evidence relations, and task support in a form that a teacher can inspect.

The risks of GenAI material generation are also material-specific. Generated versions may add unsupported explanations, flatten hedging, replace disciplinary terminology with general vocabulary, or simplify the argument until the EAP task loses its academic character. Work on hallucination and academic integrity makes documentation and human review essential (Tlili et al., 2023; Cotton et al., 2024; Gustilo et al., 2024). Broader reviews of AI in higher education point to a recurring reporting problem: implementation often advances faster than evidence on validation, learning processes, and classroom use (Crompton and Burke, 2023; Baig and Yadegaridehkordi, 2024).

Recent reviews of GenAI and conversational AI in language education show that much of the evidence still centers on higher education, English-learning contexts, writing tasks, self-report outcomes, and short-term designs (Lo et al., 2024; Lee et al., 2025; Li et al., 2025). Work on AI-supported self-regulated language learning adds that learners' uptake depends on task conditions and monitoring opportunities (Chang and Sun, 2024; Mohebbi, 2025), and meta-analytic evidence reports positive average effects with variation across context, duration, interaction type, and skill focus (Wu, 2024). EAP reading studies need to identify what the generated material changed before attributing learner outcomes to AI adaptation.

### 2.4 Evidence gap for proficiency-sensitive EAP materials

The central gap concerns two forms of AI adaptation: changing the main passage and adding a support layer around it. For EAP, separating these forms prevents accessible-looking versions from being accepted when they lose stance, evidence relations, or disciplinary terminology. It also makes room for materials that support reading without producing a large drop in automated complexity indicators. Studies that rely only on readability or simplification metrics risk missing the second case.

A fuller evaluation of differentiated EAP materials therefore needs three kinds of evidence. Expert reviewers can judge academic fidelity, proficiency appropriateness, and teachability. Automated indicators can test whether the main passages differ in structural complexity. Learner outcomes and process-related indicators can show whether the support layer changes comprehension, EAP task performance, and short-term unassisted application. The 3 × 3 comparison of original, unified-AI, and differentiated-AI materials uses these evidence sources to locate what GenAI-supported adaptation changed and for whom.

## 3 Theoretical framework

### 3.1 Proficiency-sensitive reading barriers in EAP tasks

EAP reading breaks down at several linked points. Construction–integration views of comprehension link word- and sentence-level processing to the construction of a coherent representation of the text, while cognitive-load research explains how heavy local demands can reduce the resources available for integration (Kintsch, 1998; Paas et al., 2003; Sweller et al., 2011). EAP genre research adds that the representation has to include more than propositional content: learners need to recognize claims, evidence, stance, qualification, and disciplinary purpose (Swales, 2004; Hyland, 2006, 2019). Proficiency-sensitive adaptation uses proficiency as a practical design basis for selecting which reading barrier the material should address first.

For low-proficiency readers, vocabulary and syntax load is the dominant barrier. Technical terms, nominalizations, embedded clauses, and dense sentence structures slow local processing and make it hard to connect propositions into claim–evidence relations (Grabe, 2009; Nation, 2013). The corresponding support layer uses glosses, short paraphrases, sentence unpacking, and clause segmentation. Cognitive load is the process-related indicator for this pathway because it measures perceived mental effort, rereading demand, and difficulty maintaining whole-text meaning during reading. The expected outcome is strongest for reading comprehension, where access to the main passage is a prerequisite for answering text-based questions.

Intermediate readers often cross the lexical threshold and still lose the discourse map. They may understand a sentence and still miss whether it introduces a claim, develops evidence, qualifies a position, signals contrast, or elaborates a warrant. The corresponding support layer uses rhetorical labels, paragraph-function notes, transition cues, and claim–evidence outlines. Discourse awareness is the process-related indicator because it measures recognition of claims, evidence relations, paragraph functions, and transitions. The expected outcome is strongest for EAP task performance, which asks learners to summarize, outline, and use evidence in a way that reflects the organization of the source text.

High-proficiency readers require a different support logic. They can often follow local meaning and basic organization. EAP tasks then ask them to evaluate evidence strength, interpret stance and hedging, compare alternative readings, and position their own response (Hyland, 2006, 2019). The corresponding support layer uses critical prompts, stance questions, counterclaim prompts, and questions about evidence quality. Critical engagement is the process-related indicator because it measures attention to stance, hedging, evidence strength, counterclaims, and alternative interpretations. For this group, the support layer is designed to open evaluative work without turning the response into a sequence of prescribed moves.

### 3.2 Text-embedded support and process-related indicators

Scaffolding is used here in a restricted material-design sense. The supports are fixed, text-embedded, and task-bound; they appear as glosses, labels, notes, outlines, or prompts printed in the material. Teacher mediation during reading falls outside this operationalization (Wood et al., 1976; Vygotsky, 1978; Reynolds, 2017). Table 1 maps this restricted notion of scaffolding onto four analysis elements: dominant barrier, support layer, process-related indicator, and outcome focus. It also assigns cognitive load, discourse awareness, and critical engagement to different reading problems, keeping the process variables from being treated as interchangeable.

**Table 1:** Theoretical mapping from proficiency-sensitive barriers to text-embedded supports, process-related indicators, and outcomes.

Proficiency focus	Barrier and text-embedded supports	Process-related indicator and outcome focus
Low	*Dominant barrier:* vocabulary and syntax load. *Text-embedded supports:* glosses, sentence unpacking, and clause segmentation.	*Process-related indicator:* cognitive load. *Outcome focus:* reading comprehension.
Intermediate	*Dominant barrier:* discourse navigation. *Text-embedded supports:* rhetorical labels, paragraph-function notes, and claim–evidence outlines.	*Process-related indicator:* discourse awareness. *Outcome focus:* EAP task performance.
High	*Dominant barrier:* evidence and stance evaluation. *Text-embedded supports:* critical prompts, stance questions, and counterclaim prompts.	*Process-related indicator:* critical engagement. *Outcome focus:* EAP task performance requiring evaluation or critical response.

The same mapping separates two material layers. Functional support describes what the material helps learners do: identify a term, unpack a sentence, follow a transition, connect evidence to a claim, or evaluate stance. Structural complexity describes measurable linguistic properties of the main passage, including lexical density, average sentence length, syntactic complexity, academic vocabulary coverage, cohesion markers, and hedging markers. The present study tests whether differentiated-AI versions differ at the passage level, the support-layer level, or both. Immediate unsupported application adds one further task: after supported reading, learners complete a new passage without glosses, labels, or prompts, giving a short-term check on unassisted performance.

### 3.3 Analytical framework

**Figure 1:**
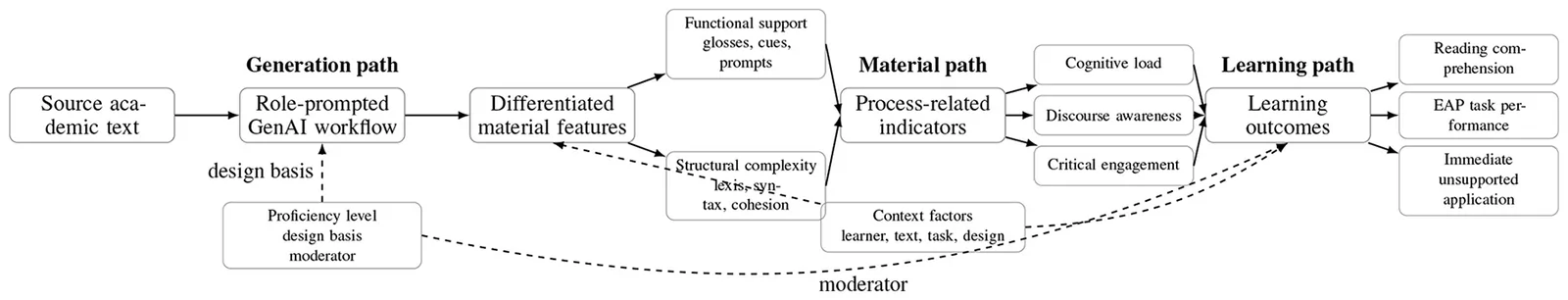
Analytical framework for judging GenAI-supported EAP material adaptation. The material path separates passage-level structural complexity from text-embedded functional support; the learning path links support types to outcomes and process-related indicators; proficiency level functions as both the design basis for differentiated materials and the moderator in the outcome models.

Figure 1 organizes the analytical judgments used to evaluate GenAI-supported adaptation. The material path separates passage-level structural complexity from the support layer so that automated metrics do not treat glosses, labels, or prompts as simplification of the main text. The learning path connects support types to reading comprehension, EAP task performance, supplementary process indicators, and immediate unsupported application. Proficiency has two roles: it guides the support selected for differentiated-AI materials and it defines the strata for learner-outcome comparisons. The omnibus Proficiency × Material term locates whether outcome patterns vary across proficiency strata. The planned within-proficiency contrasts provide the main learner-outcome comparison because they compare unified-AI and differentiated-AI materials within the same proficiency level.

Each analysis targets a different link in Figure 1. Expert ratings examine the academic fidelity, level fit, and teachability of the generated support layer. Automated complexity indicators examine the main passage after headings, task instructions, glosses, labels, and prompts have been removed. Within-proficiency contrasts compare the proficiency-specific material with the unified AI-adapted material, and two-way ANOVA models describe the wider pattern across strata. Cognitive load, discourse awareness, and critical engagement are used as supplementary process indicators that describe learner responses to the assigned support type during the reading session.

## 4 Methods

### 4.1 Design, participants, and assignment

The evaluation used a 3 × 3 between-subjects design. The first factor was proficiency level: low, intermediate, and high. The second factor was material condition: original, unified-AI, and differentiated-AI. The design compared whether a single AI-adapted version and proficiency-specific AI-adapted versions produced different outcome patterns across learner levels.

Participants were adult undergraduate EAP learners recruited from intact EAP reading classes at the author's institution. Eligibility required current enrollment in the EAP reading course, completion of the pre-study placement test, no prior exposure to the experimental reading passages, and completion of all outcome tasks. A total of 142 learners initially participated. After proficiency classification, learners were assigned at the individual level within each proficiency stratum; intact class, teacher, and timetable slot did not define condition. Seven cases were excluded before analysis: three for incomplete outcome data, two for implausibly short completion time, one for duplicate submission, and one for failing the attention-check item. The final dataset contained *N* = 135 de-identified cases, with *n* = 15 in each Proficiency × Material cell. The balanced nine-cell matrix was set before analysis; no a priori power analysis was conducted, no imputation was applied, and cases with missing primary outcome scores were excluded listwise.

Participant protection and data handling were built into the course-embedded evaluation procedure. Under the institution's regulations for minimal-risk course-based educational evaluation, formal ethics committee review was not required. Students received a written information sheet and signed written informed consent forms before participation. Participation was voluntary, withdrawal carried no penalty, and course grades, teacher evaluation, and access to learning activities were unaffected. The project collected no identifiable or sensitive personal information beyond what was needed for learning-outcome analysis. The analytic dataset contained only de-identified participant codes, proficiency group, material condition, outcome scores, and scale scores.

Learner proficiency was classified with a 60-item EAP reading placement test administered one week before the experiment. The test was scored on a 0–100 scale and showed acceptable internal consistency, *KR*-20 = 0.86. To form balanced groups for the 3 × 3 design, learners were divided by tertile cutoffs: low proficiency = 42–60 (*M* = 52.4, *SD* = 4.7), intermediate proficiency = 61–75 (*M* = 68.2, *SD* = 3.8), and high proficiency = 76–93 (*M* = 83.5, *SD* = 4.5), with *n* = 45 in each proficiency group. The effective sample retention rate was 95.1%.

**Figure 2:**
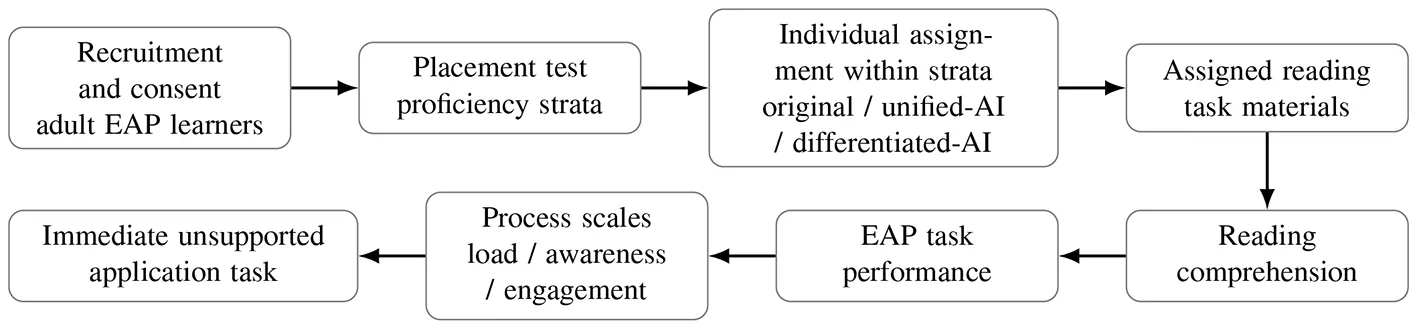
Implementation sequence for participant classification, material assignment, reading, outcome measurement, process scales, and immediate unsupported application.

Figure 2 shows the order of assignment and measurement. Proficiency classification preceded condition assignment; learners then completed the assigned reading task, reading-comprehension items, EAP task, process scales, and immediate unsupported application task. Because the process scales were collected after the main reading activity and close to the outcome measures, process-association models were used as supplementary analyses of the observed outcome pattern.

Table 2 defines the material factor used in the 3 × 3 design. Original materials retained the unmodified academic text; unified-AI materials used one AI-adapted version for all learners; differentiated-AI materials used proficiency-specific versions generated by the role-prompted workflow. Interactions involving the differentiated-AI condition test the planned match between material version and proficiency group.

**Table 2:** Material conditions used in the 3 × 3 evaluation.

Condition	Material form	Design purpose
Original	Unmodified academic text	Baseline academic fidelity and unsupported reading demand
Unified-AI	One AI-adapted version for all learners	General accessibility across proficiency levels
Differentiated-AI	Separate AI-adapted versions by proficiency	Barrier-matched support for low, intermediate, and high proficiency groups

### 4.2 Material generation workflow

**Figure 3:**
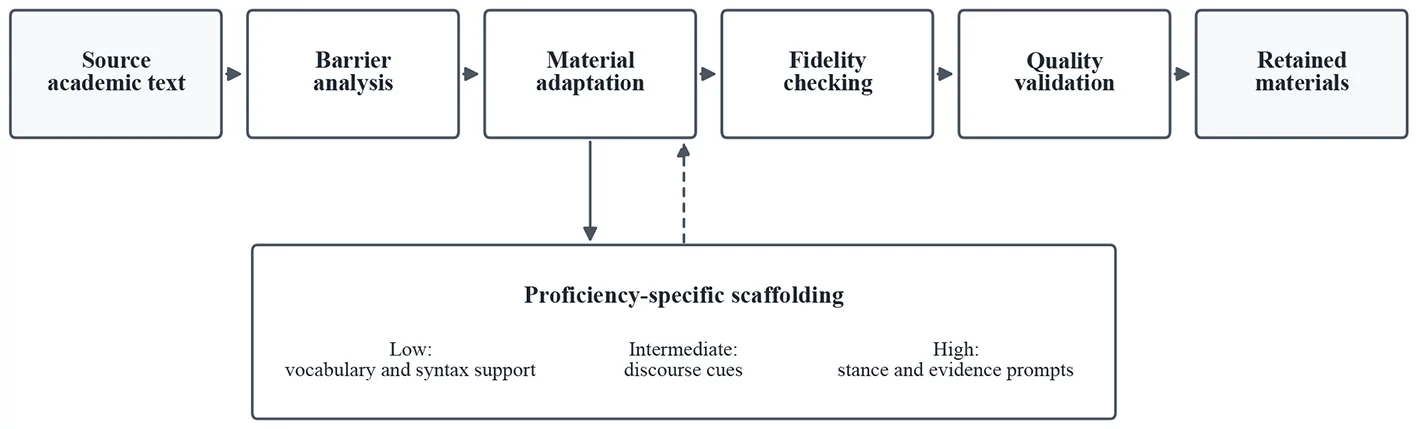
Role-prompted workflow for generating proficiency-sensitive EAP materials. Each module has a specified function: identifying barriers, adapting the text, checking academic fidelity, and validating pedagogical fit.

Figure 3 details the role-prompted GenAI workflow used to generate the support layer. Each module had a defined input, output, and human review point. The barrier-analysis module took the source passage and target proficiency profile as input and returned a list of likely reading barriers. The adaptation module used that list to generate a material version containing the relevant text-embedded supports, such as glosses, sentence unpacking, rhetorical labels, or critical prompts. The academic-fidelity module checked terminology, stance, hedging, citation or evidence relations, and argument structure against the source passage. The quality-validation module checked coherence, target-level fit, task alignment, and teachability before a version was retained or regenerated.

The workflow was implemented through the OpenAI API using GPT-4o-2024-08-06. Materials were generated between 18 and 21 April 2026 with fixed decoding settings: temperature = 0.30, top_p = 0.90, maximum output tokens = 2,500, frequency penalty = 0, and presence penalty = 0. For each source passage, one unified-AI version and three proficiency-specific differentiated versions were generated. Across the project, 12 final AI-adapted materials were retained. Nineteen generated outputs were produced in total; seven were discarded because they over-simplified academic stance, added unsupported content, or failed to match the intended proficiency level. The maximum number of regenerations for a single version was two. Human edits were limited to formatting, typographical correction, removal of unsupported additions, and alignment of task instructions. The mean proportion of edited tokens in retained materials was 4.6% (range = 1.8%–8.9%).

Table 3 illustrates how the support layer differed across versions using the cellular-respiration material. Low-proficiency support created lexical entry points through inline glosses and process breakdowns. Intermediate support mapped discourse organization through paragraph-function labels, rhetorical-move labels, claim–evidence relationships, and structural outlines. High-proficiency support shifted the reading activity toward evaluative stance work through evidence-quality, counterargument, and critical-response prompts. The same adaptation logic was applied across the source passages.

**Table 3:** Examples of differentiated material features from one source passage.

Version	Main support type	Illustrative material feature
Low	Lexical and sentence-level access	Inline support such as “glucose = a type of sugar,” “ATP is what cells use for energy,” and process breakdowns listing stages of glycolysis.
Intermediate	Discourse navigation	Paragraph-function labels, rhetorical-move labels, structural outlines, and claim–evidence notes such as “Claim: Gradual energy harvest is more efficient” linked to its evidence and warrant.
High	Argumentative evaluation	Stance and evidence prompts such as evaluating the power-plant analogy, questioning the claim that gradual energy harvest is more efficient, and considering counterexamples to redox-based energy extraction.

### 4.3 Variables and measures

Table 4 defines the measures used to evaluate material quality, learner outcomes, process-related indicators, and structural complexity. Reading comprehension measured understanding of the assigned main passage and consisted of 10 items scored from 0 to 2, yielding a 0–20 score. Two raters scored constructed-response items, and score reliability was *ICC*(2, *k*) = 0.90. EAP task performance measured academic task execution with a four-criterion rubric covering summary accuracy, claim–evidence use, rhetorical-structure recognition, and critical response; each criterion contributed up to five points, yielding a 0–20 scale. Rubric reliability was *ICC*(2, *k*) = 0.87. Immediate unsupported application measured short-term unassisted performance after the support layer was removed: learners read an unseen academic passage with no glosses, labels, or prompts and received a 0–20 score, with reliability of *ICC*(2, *k*) = 0.88.

**Table 4:** Variables and measures in the current evaluation.

Variable	Type	Range or coding	Role	Interpretation
Proficiency level	Categorical	Low 42–60; intermediate 61–75; high 76–93	Between-subjects factor and moderator	60-item EAP reading placement test; tertile split; *KR*-20 =.86
Material condition	Categorical	Original, unified-AI, differentiated-AI	Between-subjects factor	Material version assigned to learners
Reading comprehension	Continuous	10 items scored 0–2 each; 0–20	Primary outcome	Understanding of the assigned reading text; two raters for constructed-response items; *ICC*(2, *k*) = 0.90
EAP task performance	Continuous	4 criteria × 5 points; 0–20	Secondary outcome	Summary accuracy, claim–evidence use, rhetorical-structure recognition, and critical response; *ICC*(2, *k*) = 0.87
Immediate unsupported application	Continuous	Immediate unsupported text; 0–20	Unsupported application outcome	Unseen academic passage after the main reading task; no glosses, labels, or prompts; *ICC*(2, *k*) = 0.88
Cognitive load	Continuous	4 items; 4–20	Low-proficiency process indicator	Perceived mental effort or difficulty during reading; α = 0.86, ω = 0.87
Discourse awareness	Continuous	4 items; 4–20	Intermediate-proficiency process indicator	Recognition of rhetorical structure, paragraph function, and claim-evidence relations; α = 0.88, ω = 0.89
Critical engagement	Continuous	4 items; 4–20	High-proficiency process indicator	Evaluation of stance, evidence, counterclaims, and interpretation; α = 0.83, ω = 0.85
Lexical density	Continuous text metric	POS-based content-word proportion; 0–1	Structural complexity indicator	Content-word tokens divided by total lexical tokens
Sentence length	Continuous text metric	Words per sentence	Structural complexity indicator	Average sentence length in the material
Syntactic complexity	Continuous text metric	Clauses per sentence or equivalent	Structural complexity indicator	Degree of clausal embedding or sentence structure complexity
Cohesion markers	Continuous text metric	Markers per 100 words	Cohesion indicator	Frequency of explicit connectives or relation markers
Hedging markers	Continuous text metric	Markers per 100 words	Academic fidelity indicator	Frequency of epistemic qualification and stance-related expressions
Academic fidelity	Rating	1–5	Material quality indicator	Preservation of disciplinary meaning, genre features, stance, and evidence relations
Proficiency appropriateness	Rating	1–5	Material quality indicator	Match between material support and intended learner level
Teachability	Rating	1–5	Material quality indicator	Pedagogical usability for EAP instruction

The three process-related indicators were measured immediately after the reading task using four-item self-report scales. Each item was rated from 1 = strongly disagree to 5 = strongly agree, and scale scores were summed, producing a range of 4–20. Higher scores indicated higher cognitive load, stronger discourse awareness, or stronger critical engagement. Cognitive-load items assessed perceived mental effort, sentence-level difficulty, rereading demand, and difficulty maintaining whole-text meaning. Discourse-awareness items assessed recognition of claims, evidence relations, paragraph functions, and transitions. Critical-engagement items assessed attention to stance, hedging, evidence strength, counterclaims, and possible alternative interpretations. Internal consistency was acceptable for cognitive load (α* =* 0.86, ω* =* 0.87), discourse awareness (α* =* 0.88, ω* =* 0.89), and critical engagement (α* =* 0.83, ω* =* 0.85).

### 4.4 Text complexity and material quality indicators

Structural-complexity analysis tested whether differentiated versions changed the main passage itself. Objective text measures included lexical density, average sentence length, syntactic complexity, academic vocabulary coverage, cohesion markers, and hedging markers. Text complexity was analyzed in Python 3.11.6 using spaCy 3.7.2 with the en_core_web_sm 3.7.1 model for tokenization, sentence segmentation, part-of-speech tagging, lemmatization, and dependency parsing. Academic vocabulary coverage was calculated as the proportion of tokens matching an Academic Word List-based dictionary after lemmatization. Lexical density was computed as the proportion of nouns, lexical verbs, adjectives, and adverbs among all lexical tokens. Average sentence length was calculated as words per sentence. Syntactic complexity was represented by clauses per sentence, estimated from parser-based clausal dependency labels. Cohesion markers were counted using a predefined dictionary of 82 additive, contrastive, causal, and sequential connectives and reported per 100 words. Hedging markers were counted using a predefined list of 62 modal verbs, epistemic verbs, epistemic adverbs, and stance-related adjectives and reported per 100 words. Preprocessing removed headings, page numbers, references, comprehension questions, task instructions, prompt labels, glosses, rhetorical labels, and critical prompts. This preprocessing kept the metrics focused on passage-level structure and excluded support-layer density.

The separability analysis treated each material version as the unit of analysis. The target label was the intended differentiated version: low-, intermediate-, or high-proficiency. The predictors were the six *z*-standardized automated complexity indicators: lexical density, average sentence length, clauses per sentence, academic vocabulary coverage, cohesion markers per 100 words, and hedging markers per 100 words. Classification performance was estimated with leave-one-out cross-validated linear discriminant analysis. For the balanced three-label task, 33.3% served as the chance benchmark.

Expert review evaluated the support layer as a pedagogical object. Three independent reviewers rated academic fidelity, proficiency appropriateness, and teachability. All reviewers had graduate-level training in applied linguistics, TESOL, or EAP and at least 5 years of tertiary EAP teaching or materials-development experience. None participated in prompt writing, material generation, or learner-outcome scoring. Materials were anonymized and presented in randomized order. Reviewers were blind to the study hypotheses and material condition; the intended proficiency label was provided only for judging proficiency appropriateness. Reviewers used a five-point rubric, where one indicated poor fit and five indicated excellent fit. Before rating, they completed a 40-mins calibration session using three practice materials outside the final dataset. Inter-rater reliability was estimated using a two-way random-effects, absolute-agreement, average-measures ICC, *ICC*(2, *k*).

### 4.5 Statistical analysis

Main outcome models used two-way analysis of variance with proficiency, material condition, and their interaction as the omnibus model for outcome heterogeneity. For participant *k* in proficiency level *i* and material condition *j*, Equation 1 defines the model.


$$\begin{array}{l} Y_{ijk}=\mu +\alpha_{i}+\beta_{j}+{\left(\alpha \beta \right)}_{ij}+\varepsilon_{ijk}. \end{array}$$ (1)

Separate models were estimated for reading comprehension, EAP task performance, and immediate unsupported application. The interaction term was retained to describe whether material-response patterns varied across proficiency groups.

Within-proficiency planned contrasts compared differentiated-AI with unified-AI using Equation 2. These contrasts were treated as the focal learner-outcome comparisons because they held proficiency constant and compared the proficiency-specific material with the unified AI-adapted material within each stratum.


$$\begin{array}{l} C_{i}={Ȳ}_{i,\text{Diff}}-{Ȳ}_{i,\text{Unified}}. \end{array}$$ (2)

The unsupported-application check compared differentiated-AI with original materials within each proficiency level using Equation 3.


$$\begin{array}{l} P_{i}={Ȳ}_{i,\text{Diff}}^{U}-{Ȳ}_{i,\text{Original}}^{U}, \end{array}$$ (3)

where *Y*^*U*^ denotes immediate unsupported application. Values below zero with inferential support indicated lower differentiated-AI performance after visible supports were removed.

Effect sizes for planned contrasts were reported as Cohen's *d*, computed with Equation 4.


$$\begin{array}{l} d=\frac{{Ȳ}_{1}-{Ȳ}_{2}}{s_{p}},\;s_{p}=\sqrt{\frac{\left(n_{1}-1\right)s_{1}^{2}+\left(n_{2}-1\right)s_{2}^{2}}{n_{1}+n_{2}-2}}. \end{array}$$ (4)

Partial eta squared for ANOVA effects was computed with Equation 5.


$$\begin{array}{l} \eta_{p}^{2}=\frac{SS_{\text{effect}}}{SS_{\text{effect}}+SS_{\text{error}}}. \end{array}$$ (5)

Supplementary process-association models used a linear product-estimate framework with 5,000 bootstrap samples (Hayes, 2017). Within each proficiency level, material condition was treated as *X*, the process-related indicator as *M*, and the relevant learning outcome as *Y*. Equations 6–8 define the product estimates.


$$\begin{array}{l} M=i_{M}+aX+e_{M}, \end{array}$$ (6)

$$\begin{array}{l} Y=i_{Y}+c^{\prime }X+bM+e_{Y}, \end{array}$$ (7)

$$\begin{array}{l} \text{Product estimate}=ab. \end{array}$$ (8)

Bootstrap confidence intervals crossing zero were interpreted as non-significant. Because the process indicators and learning outcomes were collected in the same session and close in time, the product estimates were reported as supplementary process-related associations.

## 5 Results

### 5.1 Material quality and complexity

The first analysis examined whether the generated versions were acceptable as EAP materials and whether their differentiation appeared in passage-level structural metrics. Three independent EAP reviewers evaluated 15 anonymized material versions. They rated the materials favorably for academic fidelity (*M* = 4.24, *SD* = 0.41), proficiency appropriateness (*M* = 4.36, *SD* = 0.38), and teachability (*M* = 4.31, *SD* = 0.44); the overall quality mean was *M* = 4.30 (*SD* = 0.37). Inter-rater reliability was acceptable, *ICC*(2, *k*) = 0.84, 95% CI [0.76, 0.91], using a two-way random-effects, absolute-agreement, average-measures model. These ratings indicate that reviewers accepted the versions as usable EAP materials that preserved academic meaning, fitted the intended level, and could be taught in a reading class. The ratings do not merely indicate that the passages became easier to read.

The automated indicators addressed whether the low-, intermediate-, and high-proficiency versions formed distinct structural profiles in the main passages. Across lexical density, sentence length, clauses per sentence, academic vocabulary coverage, cohesion markers, and hedging markers, the differentiated versions showed limited structural separation. The six selected indicators yielded $\eta_{p}^{2}=0.043$, and the leave-one-out classification accuracy was 11.1%, compared with the 33.3% benchmark for the balanced three-label task. These results show that the differentiated versions did not form a stable passage-level structural gradient. The material distinction lies outside a simple progression from easier text to harder text.

**Figure 4:**
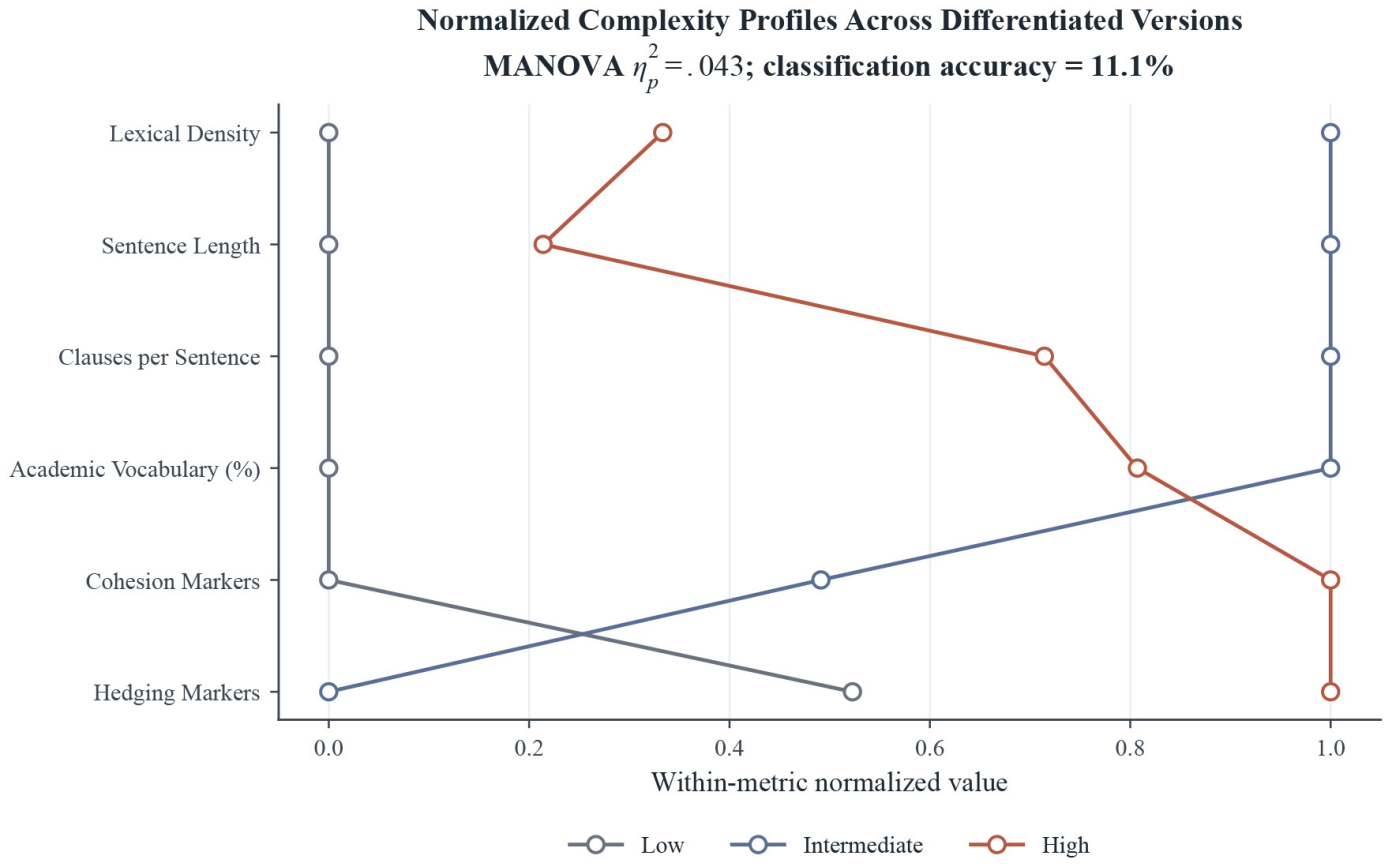
Normalized complexity profiles across differentiated versions. Values are normalized within each metric. The selected structural metrics showed limited separation across the three versions.

Figure 4 shows why the automated indicators did not classify the intended version labels. The normalized curves do not produce a consistent easier-to-harder sequence from low to high proficiency. Academic vocabulary coverage, hedging markers, and cohesion markers do not decrease or increase systematically with the intended proficiency label. Sentence length and syntactic complexity also fail to mark a clear three-level progression. The selected automated metrics therefore cannot, on their own, identify the differentiated versions.

This result should be read alongside the material examples in Table 3. Figure 4 indicates that the main passages remained close in measurable structural profile, while Table 3 shows where the differentiated design appeared: low-proficiency versions added glosses, sentence unpacking, and process breakdowns; intermediate versions added rhetorical labels, paragraph-function notes, and claim–evidence mapping; high-proficiency versions added prompts for stance, evidence quality, counterargument, and critical response. The material adaptation changed the work that learners were asked to do with the passage.

Table 5 places the two forms of material evidence side by side. Expert ratings record reviewers' acceptance of the generated versions as academically faithful, level-appropriate, and teachable EAP materials. Automated indicators record the small amount of passage-level structural separation. The combination directs attention to text-embedded functional support: glosses, sentence unpacking, rhetorical labels, claim–evidence notes, and critical prompts explain the differentiated versions more clearly than broad simplification of the main passage.

**Table 5:** Material quality and complexity indicators in the current evaluation.

Indicator	Target or benchmark	Observed value	Interpretation
Academic fidelity rating	>4.00	*M* = 4.24, *SD* = 0.41	Favorable expert rating
Proficiency appropriateness	>4.00	*M* = 4.36, *SD* = 0.38	Favorable expert rating
Teachability	>4.00	*M* = 4.31, *SD* = 0.44	Favorable expert rating
Overall material quality	>4.00	*M* = 4.30, *SD* = 0.37	Favorable overall rating
Inter-rater reliability	*ICC*>0.75	*ICC*(2, *k*) = 0.84, 95% CI [0.76, 0.91]	Acceptable reliability
Complexity MANOVA	$\eta_{p}^{2}>0.14$	$\eta_{p}^{2}=0.043$	Limited structural differentiation
Classification accuracy	>33.3% benchmark for balanced three-label task	11.1%	Limited structural separation

### 5.2 Reading comprehension and EAP task performance

The clearest learner-outcome evidence comes from the within-proficiency planned contrasts in Table 6, where proficiency is held constant and differentiated-AI is compared directly with unified-AI. For low-proficiency learners, differentiated-AI exceeded unified-AI by 1.56 points (13.23 vs. 14.79), *d* = 0.60. For intermediate learners, the difference was 0.38 points (14.55 vs. 14.92), *d* = 0.16. For high-proficiency learners, the difference was 2.47 points (16.26 vs. 18.73), *d* = 1.40. This within-stratum comparison identifies the high-proficiency group as the strongest case for added value beyond the unified AI-adapted material, followed by a moderate low-proficiency advantage and a small intermediate difference.

**Table 6:** Planned contrasts comparing differentiated-AI with unified-AI on reading comprehension.

Proficiency	Unified-AI mean	Differentiated-AI mean	Mean difference	Cohen's *d*
Low	13.23	14.79	1.56	0.60
Intermediate	14.55	14.92	0.38	0.16
High	16.26	18.73	2.47	1.40

The three contrasts describe different forms of material uptake. In the low-proficiency group, the moderate advantage is consistent with local access support: glosses, sentence unpacking, clause segmentation, and process breakdowns helped learners enter the passage and answer comprehension items. In the intermediate group, differentiated discourse cues added little over the unified-AI version. General readability support may already have met part of this group's reading need, and paragraph-function labels or claim–evidence outlines did not translate into a large comprehension gain in the current task. In the high-proficiency group, the large advantage indicates that advanced readers responded most strongly to stance-, evidence-, and counterargument-oriented support. The unified-AI version gave general accessibility support, but the differentiated version better matched the evaluative work expected of advanced EAP readers.

**Figure 5:**
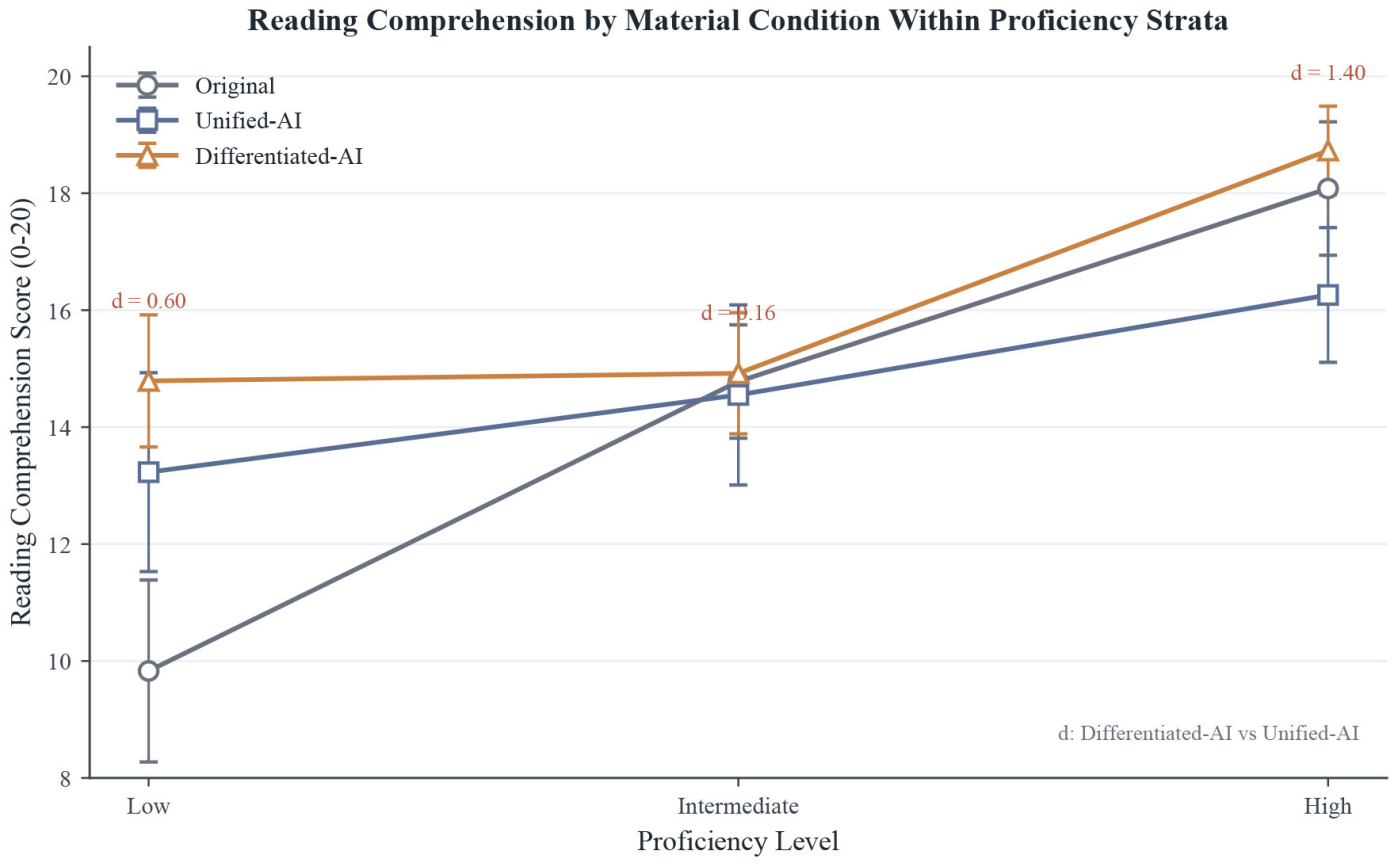
Reading comprehension by material condition within proficiency strata. Error bars represent 95% confidence intervals. Effect-size labels compare differentiated-AI with unified-AI within each proficiency level.

Figure 5 places these planned contrasts in the full three-condition pattern. The low-proficiency line shows the original condition as the weakest point and the differentiated-AI condition above unified-AI. The intermediate line is comparatively flat, matching the small contrast in Table 6. The high-proficiency line shows the largest separation between differentiated-AI and unified-AI, with differentiated-AI reaching the highest mean in the figure. The visual pattern is therefore consistent with the planned contrasts while also showing where the original materials sit within each proficiency stratum.

The omnibus ANOVA was consistent with this stratified pattern. As shown in Table 7, reading comprehension varied by proficiency, F_(2, 126)_ = 56.81, *p* < 0.001, and $\eta_{p}^{2}=0.474$, material condition, F_(2, 126)_ = 8.81, *p* < 0.001, and $\eta_{p}^{2}=0.123$, and Proficiency × Material, F_(4, 126)_ = 7.43, *p* < 0.001, and $\eta_{p}^{2}=0.191$. The interaction term located heterogeneity across proficiency strata, while Table 6 specifies the differentiated-AI vs. unified-AI contrasts within each stratum. Table 7 also shows that immediate unsupported application was organized by proficiency rather than material condition, which keeps the learner-outcome interpretation centered on supported reading performance.

**Table 7:** Summary of ANOVA results for reading comprehension, EAP task performance, and immediate unsupported application.

Outcome	Effect	*F*	*p*	$\eta_{p}^{2}$
Reading comprehension	Proficiency	56.81	< 0.001	0.474
Reading comprehension	Material	8.81	< 0.001	0.123
Reading comprehension	Proficiency × Material	7.43	< 0.001	0.191
EAP task performance	Proficiency	61.53	< 0.001	0.494
EAP task performance	Material	7.20	0.001	0.103
EAP task performance	Proficiency × Material	3.01	0.021	0.087
Immediate unsupported application	Proficiency	45.28	< 0.001	0.418
Immediate unsupported application	Material	0.82	0.445	0.013
Immediate unsupported application	Proficiency × Material	0.31	0.870	0.010

For EAP task performance, Table 7 reports the same general direction with smaller effects: proficiency, F_(2, 126)_ = 61.53, *p* < 0.001, and $\eta_{p}^{2}=0.494$; material, F_(2, 126)_ = 7.20, *p* = 0.001, and $\eta_{p}^{2}=0.103$; and Proficiency × Material, F_(4, 126)_ = 3.01, *p* = 0.021, and $\eta_{p}^{2}=0.087$. This weaker interaction fits the nature of the outcome. Reading-comprehension items primarily assess understanding of the assigned passage, whereas EAP task performance requires summary accuracy, claim–evidence use, rhetorical-structure recognition, and critical response in a produced answer. The support layer was more visible in immediate passage comprehension than in rubric-scored academic task production.

### 5.3 Supplementary process-related analyses

The supplementary process-related analyses describe how learners reported their reading experience under the assigned support type. For low-proficiency learners, differentiated materials were associated with lower cognitive load, *a* = −2.54, *p* < 0.001, and the product estimate was *ab* = −0.18, 95% CI [−1.67, 1.89]. Learners reported less mental effort, less sentence-level difficulty, and less rereading pressure after using the low-proficiency support layer. The product estimate crossed zero, so the process result is reported as a descriptive signal about perceived load during supported reading.

For intermediate learners, differentiated materials were associated with higher discourse awareness, *a* = 1.36, *p* < 0.001, and the product estimate was 0.16, 95% CI [−0.58, 0.90]. Learners reported greater awareness of claims, paragraph functions, and evidence relations after using the discourse cues. The product estimate crossed zero, matching the small differentiated-AI advantage observed in the within-proficiency reading-comprehension contrast.

**Figure 6:**
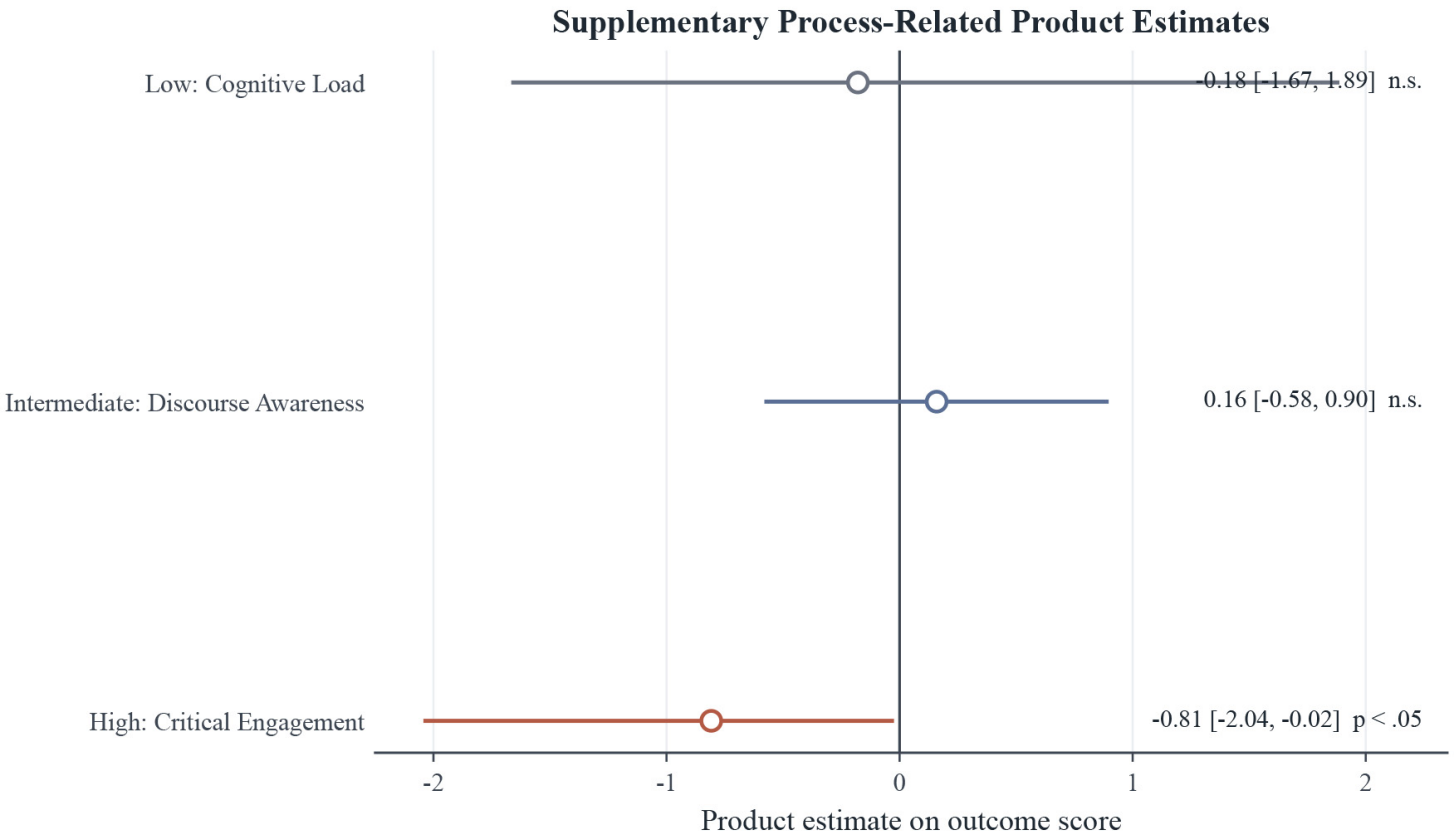
Supplementary process-related product estimates. Product estimates are reported descriptively because process indicators and outcomes were measured in the same session.

For high-proficiency learners, the product estimate involving critical engagement and EAP task performance was negative, *ab* = −0.81, 95% CI [−2.04, −0.02]. The estimate is reported as a concurrent process pattern from a small cell size. One hypothesis for future material-design studies is that advanced-reader prompts may need to support integrated evaluation rather than separate checklist responses. The present data raise this possibility without establishing it as a mediated mechanism. Figure 6 summarizes the three product estimates.

Figure 6 and Table 8 place the process indicators next to the learner-outcome pattern without treating them as a causal chain. Low-proficiency support corresponded to lower perceived load, intermediate support corresponded to greater discourse awareness, and the high-proficiency estimate raises a testable question about how advanced readers respond to different prompt formats.

**Table 8:** Supplementary process-related model estimates.

Proficiency	Processindicator	*X*–processassociation	Process–outcomeassociation	Product estimate [95% CI]
Low	Cognitive load	−2.54^***^	0.07	−0.18 [−1.67, 1.89], n.s.
Intermediate	Discourse awareness	1.36^***^	0.12	0.16 [−0.58, 0.90]
High	Critical engagement	1.13^**^	−0.71^*^	−0.81 [−2.04, −0.02]

*X* denotes material condition within each proficiency level. ^*^p < 0.05, ^**^p < 0.01, and ^***^p < 0.001. Product estimates are statistical *ab* estimates with bootstrap confidence intervals and are reported descriptively.

### 5.4 Immediate unsupported application

**Figure 7:**
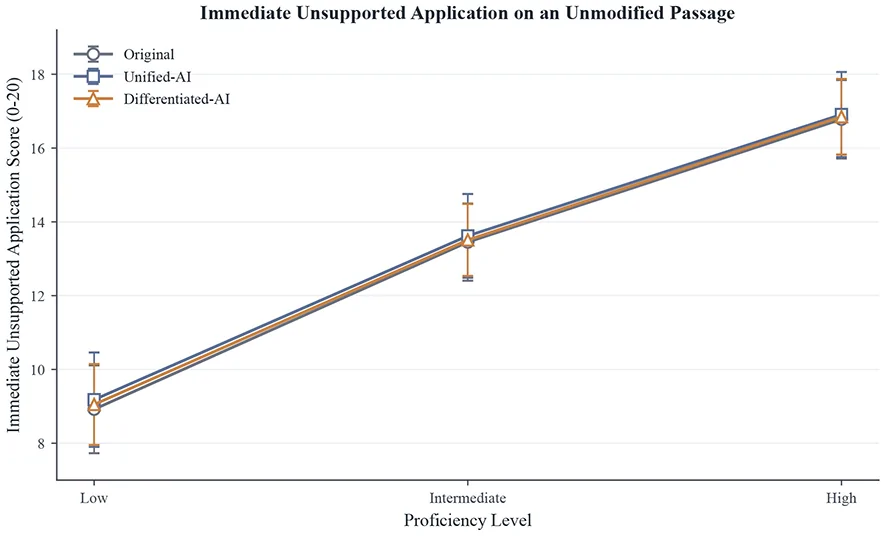
Immediate unsupported application on an unmodified passage. Error bars represent 95% confidence intervals. Omnibus statistics showed a non-significant material effect and a non-significant Proficiency × Material interaction.

Immediate unsupported application tested short-term unassisted performance on a new academic passage after glosses, labels, and prompts had been removed. The task showed a significant proficiency effect, F_(2, 126)_ = 45.28, *p* < 0.001, and $\eta_{p}^{2}=0.418$, indicating that unsupported application differed across proficiency levels. The material effect was non-significant, F_(2, 126)_ = 0.82, *p* = 0.445, and $\eta_{p}^{2}=0.013$, and the Proficiency × Material interaction was also non-significant, F_(4, 126)_ = 0.31, *p* = 0.870, and $\eta_{p}^{2}=0.010$. Performance on this task was organized mainly by proficiency level. Figure 7 presents the group means for the unassisted passage.

Figure 7 shows no visible short-term dependence cost after support removal. Differentiated-AI means were not lower than original-material means within the three proficiency groups, and the planned unsupported-application comparisons were non-significant. In this immediate task, differentiated-AI support did not produce a performance drop on a new unmodified passage. Because learners completed the task in the same session, the result concerns immediate unassisted performance; delayed transfer and strategy internalization remain separate outcomes for later classroom work.

## 6 Discussion

The present data support an interpretation centered on support-layer design. Expert reviewers rated the generated versions as academically faithful, level-appropriate, and teachable, and the automated complexity indicators did not separate the low-, intermediate-, and high-proficiency versions into a stable passage-level sequence. The clearest learner-outcome evidence came from the planned contrasts comparing differentiated-AI with unified-AI within each proficiency stratum. This ordering matters for the interpretation of GenAI-supported adaptation: the differentiated materials varied most in the forms of help placed around the academic passage, and the strongest learning signal appeared when those forms of help were compared with a single AI-adapted version for learners at the same proficiency level.

Academic fidelity and structural complexity measure different requirements in EAP reading materials. EAP materials cannot be judged by readability alone because hedging, citation relations, technical terminology, and claim–evidence structure carry the academic work that learners need to practice. A version that removes these features may improve surface accessibility while weakening the genre and discourse demands of the task. The expert ratings show that the generated versions retained enough disciplinary meaning, stance, evidence relations, and teachability to function as EAP materials. The weak structural separation then becomes informative: the workflow kept the main passages close enough in measurable form and added support that helped learners enter, map, or evaluate the academic argument.

For low-proficiency learners, the moderate differentiated-AI advantage over unified-AI fits the local-access function of the support layer. Glosses, sentence unpacking, clause segmentation, and process breakdowns provide entry points into the passage before learners answer comprehension items. The process estimates are consistent with this reading because low-proficiency learners reported lower cognitive load under differentiated-AI materials. The learning result is therefore best understood as an immediate supported-reading gain linked to passage entry and sentence-level processing.

The intermediate group showed a small differentiated-AI advantage over unified-AI, even though the differentiated version made discourse relations more visible. This pattern suggests that paragraph-function labels and claim–evidence outlines may have overlapped with the general readability support already present in the unified-AI version. It also raises a task-alignment question for later materials work: discourse cues may need to connect more explicitly to summary construction, claim selection, and evidence use if they are expected to change rubric-scored EAP production. In the present reading-comprehension contrast, their added value was modest.

The high-proficiency planned contrast was the largest learner-outcome result. Because the comparison is made within the high-proficiency stratum, it speaks more directly to material design than the omnibus interaction term. Advanced readers appeared to benefit from support that directed attention to stance, evidence quality, counterargument, and disciplinary response. The result also broadens the role of differentiation in EAP materials: proficiency-sensitive support is not confined to reducing access barriers for lower-level learners; it can also preserve textual challenge while organizing higher-order evaluative work for advanced readers.

The supplementary process-related analyses add concurrent signals about learner experience during the same reading session. The negative product estimate for critical engagement invites future testing of how advanced-reader prompts differ when they are written as open synthesis prompts, checklist prompts, or evidence-evaluation prompts. The present design uses these estimates descriptively, so the high-proficiency outcome advantage is interpreted through the within-group material comparison rather than through a temporal process claim. Future studies can separate reading process and outcome measurement more clearly by collecting annotations, logs, think-aloud data, or delayed task responses.

The role-prompted GenAI workflow offers a practical way to make this kind of adaptation reviewable. Barrier analysis names the reading obstacle for the target group; adaptation translates that obstacle into glosses, cues, notes, or prompts; fidelity checking screens for unsupported additions, flattened stance, lost terminology, and over-simplified reasoning; validation checks level fit, coherence, teachability, and task alignment before a version is retained. The immediate unsupported application task added a short-term check after visible supports were removed. Learners who used differentiated-AI materials did not show a performance drop on the new unmodified passage in the same session, and delayed transfer, strategy internalization, and sustained classroom uptake remain questions for longer EAP reading sequences.

## 7 Limitations and future work

The evidence comes from a balanced classroom evaluation with *N* = 135 adult undergraduate EAP learners and 15 learners in each Proficiency × Material cell. This scale supports the planned comparison across proficiency and material conditions, and larger balanced samples would make the within-group contrasts and process-related estimates more stable. The course setting, short reading sequence, three source passages, and 15 reviewed material versions give a focused view of support-layer adaptation in one EAP instructional context; extensions across instructors, institutions, disciplines, genres, and delayed reading tasks would show how the same workflow performs with broader classroom and text variation. The process-related indicators were collected through post-reading self-report scales close to the outcome tasks, making them useful for learner perception and material-design feedback; reading logs, annotations, screen recordings, and interviews could capture how learners actually move between glosses, rhetorical cues, prompts, and the main passage during reading. Because GenAI outputs also depend on model release, access conditions, and prompt execution, future evaluations should continue to archive model version, generation settings, prompt modules, discarded outputs, human-edit rates, expert review records, and complexity metrics so that later comparisons remain traceable.

## 8 Conclusion

The evaluation shows that GenAI-supported EAP adaptation changed functional support more clearly than passage-level structural complexity. The strongest learner evidence came from within-proficiency contrasts, especially the high-proficiency advantage of differentiated-AI over unified-AI, with the omnibus Proficiency × Material term retained to locate heterogeneous response patterns across strata. Supplementary process estimates were descriptive and are better suited to future hypothesis testing than to mediation claims. Immediate unsupported application showed no short-term performance cost after visible supports were removed. For EAP materials work, the main lesson is that academic fidelity, support–barrier matching, and task pathway should guide GenAI adaptation alongside structural-complexity checks. The role-prompted workflow makes this work inspectable by separating barrier analysis, adaptation, fidelity checking, and validation, producing reviewable drafts that preserve disciplinary argument while varying the help learners receive as they enter, map, and evaluate academic texts.

## Data Availability

The raw data supporting the conclusions of this article will be made available by the authors, without undue reservation.
